# How Women Bathe in Paris

**Published:** 1892-05

**Authors:** 


### HOW WOMEN BATHE IN PARIS.

The Seine is a narrow, greenish, snake-like river, and it doesn't look inviting from a distance. But on entering the swimming baths formed of boat-like sides, containing the dressing-rooms, it looks clear and cool. Black bathing dresses trimmed with red braid can be hired for a franc upward, and hundred of ladies, with their children, daily afford themselves amusement at these places. There is no effort at fashion, gentlemen not admitted, and the top being covered by canvas to shield them from the eyes of people on embankment or boulevards. Swimmers among the attendants are frequent, and they dive and float, swim and sink at will, and teach the children and ladies to do the same.

Some of the French women are expert swimmers, and they go around in their black, baggy suits, chasing about the platforms, eating sandwiches or drinking penny syrups or clarets, and end the performance by diving into the water and swimming a race. Trapezes, swings, etc., are suspended above the water, and gymnastic exercises are indulged in by the younger and more ambitious.

On leaving the baths they dry their hair sufficiently, put a crimpled perruke over their own straight locks, a dash of powder and a bit of lip salve (a stick of which every French woman carries in her pocket), to slightly color and eliminate any dryness, and with a deft adjustment of the inevitable black veil, my lady is well coiffed, and, if her dress be suitable, can take a drive before going home.

### WORK WINS.

We look back into the past as a restriction of power, a restriction of liberty, and a restriction of sphere. History was the biography of the few, and the history of a people was never written till the present century. A great change has come. The chances once held by the brilliant and the strong, and other chances non-existent then, have opened out on every hand. There is work and success for the men of every kind and degree of gift; not alone room at the top, as there always was, but all the way up; not only for the best things of the best men, but for every man's best. The very perplexity in the outlook before the young man now is from the "embarrassment of riches." It appears in the ever increasing forms of material and business enterprise, in the specialties of the professions, in the reconstructions of history, in the boundless range of scientific investigation and the practical application of science to art, in linguistic and archæological researches, in the lines of Biblical investigation, in the enormous expansions of philanthropy—till we may say to every man, "What can you do? Go and do it. Whatever your gift and drift, there is a place waiting for just such a man as you; or if not, you can make such a place." The man that is ready finds the opportunity also ready. The few who make their opportunities find a Providence opening the way. Work wins. And the best kind of genius is a genius for wise, hard work.—*Ex.*

